# Antidepressive and BDNF effects of enriched environment treatment across ages in mice lacking BDNF expression through promoter IV

**DOI:** 10.1038/tp.2016.160

**Published:** 2016-09-20

**Authors:** S Jha, B E Dong, Y Xue, D F Delotterie, M G Vail, K Sakata

**Affiliations:** 1 Department of Pharmacology, University of Tennessee Health Science Center, Memphis, TN, USA

## Abstract

Reduced promoter IV-driven expression of brain-derived neurotrophic factor (BDNF) is implicated in stress and major depression. We previously reported that defective promoter IV (KIV) caused depression-like behavior in young adult mice, which was reversed more effectively by enriched environment treatment (EET) than antidepressants. The effects of promoter IV-BDNF deficiency and EET over the life stages remain unknown. Since early-life development (ED) involves dynamic epigenetic processes, we hypothesized that EET during ED would provide maximum antidepressive effects that would persist later in life due to enhanced, long-lasting BDNF induction. We tested this hypothesis by determining EET effects across three life stages: ED (0–2 months), young adult (2–4 months), and old adult (12–14 months). KIV mice at all life stages showed depression-like behavior in the open-field and tail-suspension tests compared with wild-type mice. Two months of EET reduced depression-like behavior in ED and young adult, but not old adult mice, with the largest effect in ED KIV mice. This effect lasted for 1 month after discontinuance of EET only in ED mice. BDNF protein induction by EET in the hippocampus and frontal cortex was also the largest in ED mice and persisted only in the hippocampus of ED KIV mice after discontinuance of EET. No gender-specific effects were observed. The results suggest that defective promoter IV causes depression-like behavior, regardless of age and gender, and that EET during ED is particularly beneficial to individuals with promoter IV-BDNF deficiency, while additional treatment may be needed for older adults.

## Introduction

Enriched environment treatment (EET), which includes physical exercise, mental stimulation and social interaction,^1, 2, 3^ provides neurotrophic effects and is a highly promising alternative to prevent/treat major depressive disorder.^4, 5, 6^ We recently found that chronic EET was more effective^7^ than chronic antidepressant drug treatment^8^ in reversing depression-like behavior caused by deficiency of promoter IV-driven expression of brain-derived neurotrophic factor (BDNF).^9, 10, 11, 12^ BDNF is an important neuronal growth factor involved in the pathophysiology of depression.^12, 13, 14, 15, 16, 17, 18^ Studies have shown decreased BDNF levels in the hippocampus and (pre)frontal cortex of depressed patients^19^ and stressed animals.^20, 21, 22, 23^ In particular, epigenetic inactivation of promoter IV has been reported in depressed patients^24, 25^ and stressed animals.^20, 22^ We have shown causal evidence that defective promoter IV leads to depression-like behavior^10^ using KIV mice. KIV mice lack promoter IV-driven BDNF but retain 8 other promoters and the BDNF protein-coding region.^9^ BDNF gene expression is regulated by at least 9 promoters in both humans^26^ and rodents.^27^ These multiple promoters may reflect the importance of BDNF expression, serving as backups in case of one or more promoter deficiencies. We previously tested this hypothesis using young adult mice (2–4 months old) to find that reduced BDNF expression by promoter IV defect could be compensated through other BDNF promoters by 3-week EET^7^ but not by 3-week treatments with 4 different classes of antidepressants.^8^ EET or exercise (one of EET's most important components)^28, 29, 30^ has been shown to induce BDNF via multiple promoters I, II and III^7, 31, 32, 33^ while causing epigenetic modification at promoters II, IV and VI.^34, 35^

The neurotrophic effects of EET have been extensively studied over the last 60 years.^7, 36, 37, 38, 39^ However, its antidepressive effects and neurotrophic mechanisms, particularly its age-dependent effects, remain unclear. At which stage in life is EET most effective for depression, and how? Identifying the life period when EET effects are maximal and revealing its mechanisms are important for developing strategies for effective prevention/treatment of depression. As early-life development (ED) involves dynamic gene expression regulation and long-lasting epigenetic processes,^40^ we hypothesized that antidepressive effects of EET and BDNF compensation may be maximized when EET is provided during ED, and that the effects will persist later in life due to long-lasting BDNF expression changes. Using our unique mouse model of depression that lacks promoter IV-driven BDNF expression (KIV mice),^9, 10^ here we tested our hypotheses by determining the antidepressive and BDNF effects of EET across ages: ED, young adult and old adult. We also tested whether the EET effects last after 1 month of discontinuance of EET.

## Materials and methods

### Animals

**Figure 1:**
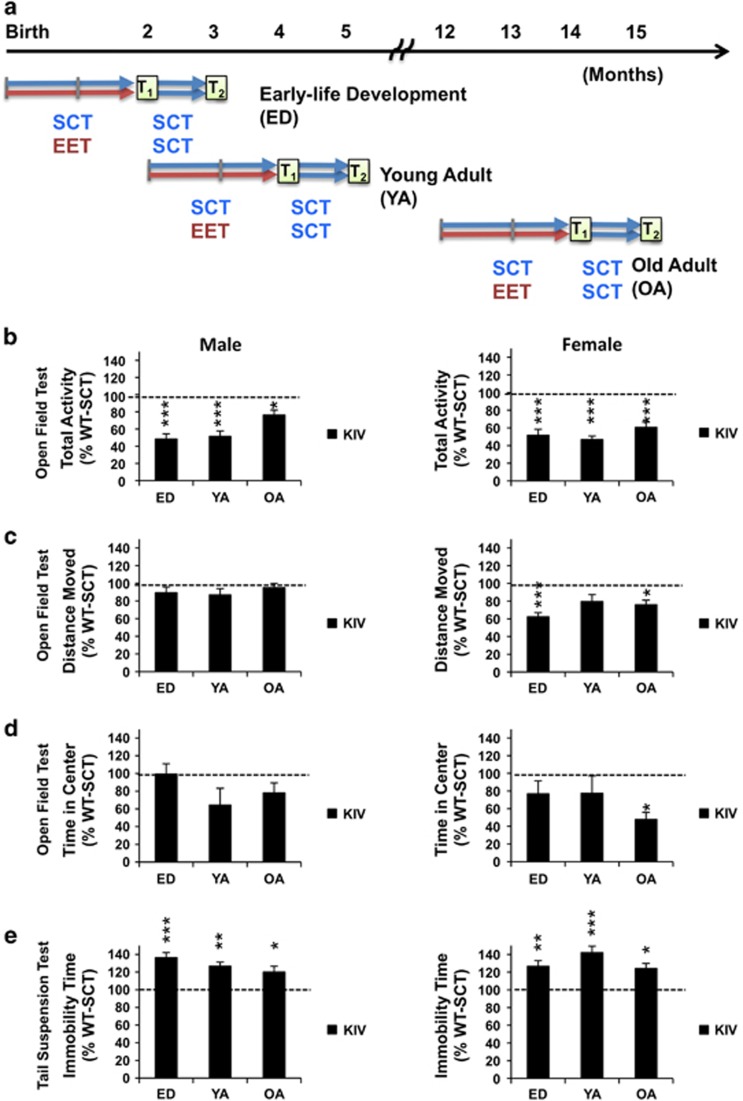
(**a**) Research design. Male and female wild type (WT) and KIV mice received 2 months of enriched environment treatment (EET: red arrows) or standard condition treatment (SCT: blue arrows) from birth (early-life development: ED), 2 months (young adult: YA) or 12 months of age (old adult: OA), then received 1 month of SCT. Depression-like behavior and brain-derived neurotrophic factor (BDNF) levels were measured after EET (test 1: T_1_) and consequent SCT (test 2: T_2_). (**b–e**) Age and gender effects of deficiency of promoter IV-driven BDNF on total activity (**b**); distance moved (**c**); and time in center (**d**) in the open-field test, and immobility time in the tail-suspension test (**e**). Results from KIV-SCT mice (male: left; female: right) at T_1_ are shown as % WT-SCT. Asterisks on the columns show a significant difference between WT and KIV mice. KIV, knock-in BDNF-promoter IV; SCT, standard condition treatment; WT, wild-type. *P<0.05, **P<0.01, ***P<0.005. *N=*12**–**16.

Wild-type (WT) and knock-in BDNF-promoter IV (KIV)^9^ mice were used to assess effects of EET in normal and depressed^10^ conditions, respectively. The generation of KIV mice has been described previously.^9^ Briefly, KIV mice were generated from 129/sv ES cells with C57BL/6 J blastocytes and crossed to C57BL/6 J females for >12 generations. Heterozygous mice were bred to produce WT and KIV littermates with the same genetic background. WT and KIV offspring from these WT and KIV littermates were used in this study. Male and female mice of both genotypes were used to examine gender-specific effects. EET effects were examined at three life stages: early-life development (ED: 0–2 months), young adult (2–4 months) and old adult (12–14 months; Figure 1a), covering a period from birth to reproductive maturity, during sexual maturity and middle-age in mice (Jax; https://www.jax.org/research-and-faculty/research-labs/the-harrison-lab/gerontology/life-span-as-a-biomarker),^41^ respectively. A cohort of mice was used to assess EET effects on depression-like behavior (total ~360 mice: *N=*10–16 per group × 2 genotypes × 2 treatment conditions × 2 genders × 3 life stages). Another cohort of mice was used to assess EET effects on BDNF protein levels (total ~240 mice: *N=*4–5 per group × 2 genotypes × 4 treatment conditions × 2 genders × 3 life stages). The sample size was chosen based on our previous experiments^7, 9, 10^ and power analyses. Mice were kept in a normal 12:12 h dark–light cycle with *ad libitum* access to food and water. All animal experiments were approved by the University of Tennessee Laboratory Animal Care and Use Committee.

### Treatments

Age- and gender-matched mice were randomly assigned to standard condition treatment (SCT) or EET. SCT consisted of a regular cage (27 × 16 × 12 cm) containing 2–5 mice (group-housed to avoid isolation stress). EET consisted of a larger cage (44 × 22 × 16 cm) containing one plastic running wheel per 5 mice to increase physical exercise, an assortment of toys (igloo, dome, balls, tunnels and so on) to increase perception/mental exercise, and 5–10 company mice with nesting material to increase social interaction. The toys were changed weekly, and mice with EET were given rodent Foraging Crumble (Bio-Serv, Frenchtown, NJ, USA). For ED-EET, pups born in EET were raised and then weaned to another EET cage at 3 weeks of age.

Mice were housed with either SCT or EET for 2 months. This period was chosen to encompass the developmental period of mice from birth to reproductive maturity. We chose chronic EET because recovery from depression usually takes at least several weeks. After 2 months of treatment, the mice were placed in standard condition for 1 month to examine whether EET effects lasted after its discontinuance (see Figure 1a for research design).

### Behavioral tests

Depression-like behavior of mice was assessed by the open-field test on day 1 and the tail-suspension test on day 2, as described previously.^7, 10^ The open-field test measures spontaneous explorative locomotor activity in a novel open field (47 × 37 × 20 cm, 900 lux in the center) for 10 min.^42^ Total activity was measured by infrared beam breaks (Accuscan Instruments, Columbus, OH, USA). Time spent in the center (25% of the field) and distance moved were measured by a video tracking system (EthoVision, Noldus, VA, USA). The tail-suspension test measures stress-related despair when mice are suspended by their tails;^43^ the presence of immobility was assessed over a 6-min session by a trained observer.^44^ These two tests were selected because they previously detected depression-like phenotypes in young adult KIV males,^7, 10^ and their short testing time minimized animal stress. Data quantification that potentially includes subjective bias (immobility measured in the tail-suspension test) was verified by an additional observer blinded to the genotype and experimental group.

### BDNF ELISA

Brain tissue from mice with 4 treatments (SCT/EET at T_1_ and SCT–SCT/EET–SCT at T_2_, see Figure 1a) were collected between 1400 and 1700 hours and kept in −80 °C freezer until processed. Protein samples were prepared from the right hippocampus and frontal cortex from each mouse, as described previously.^9^ BDNF protein levels were determined by ELISA (BDNF Emax; Promega, Madison, WI, USA) and normalized to the total protein concentrations measured by the DC protein assay (Bio-Rad, Hercules, CA, USA).

### Statistical analyses

Two-way and three-way analyses of variance were performed for detecting genotype, EET, and age effects, followed by *post hoc* Bonferroni multiple comparisons. Student's *t*-tests (two-tailed) were performed on the two data groups. Normal distribution was verified by *F*-test. Outlier data, as defined and pre-established as above/below the means±two s.d., were excluded to avoid type II errors. Data are presented as means±s.e.m. Statistical significance was set at *P<*0.05.

## Results

We examined the effect of EET on depression-like behavior and BDNF levels in WT and KIV mice during ED, young adult, and old adult stages and tested whether these effects persisted after 1 month without EET (Figure 1a). Specifically, we asked whether: (1) promoter IV-BDNF deficiency caused depression-like behavior in juvenile (ED) and old adult mice of both genders; (2) antidepressive EET effects differed among the three life stages or between genders; (3) the EET effects endured *after* 1 month; and (4) EET effects on BDNF expression correlated with the behavioral effects. Statistics are presented in Supplementary Table 1.

### Promoter IV-BDNF deficiency results in depression-like behavior, regardless of age and gender

First, we determined effects of promoter IV-BDNF deficiency on explorative activity across ages using the open-field test. Male and female KIV mice at all three life stages showed significant reductions in total activity compared with their WT counterparts (Figure 1b, at least *P*<0.05 for each, statistics in Supplementary Table 1). When distance moved was analyzed, no difference between genotypes was observed in all male groups and young adult females (Figure 1c, *P*>0.05). These results suggested that the reduction of total activity was attributed to reduced vertical activity (for example, rearing to observe outside), which is thought to reflect exploratory activity in rodents^45^ rather than agitation.

Analyses of time spent in the center, an anxiolytic indicator,^46, 47^ showed no difference between genotypes of males at all ages (Figure 1d, *P*>0.05 for each), similar to our previous result with young adult males.^7, 10^ However, females showed a significant genotype effect (*P*<0.05), where only old adult KIV females showed reduced time in the center (Figure 1d, WT-SCT vs KIV-SCT: *P*<0.05). This indicated that promoter IV-BDNF deficiency increased anxiety-like behavior only in aged females.

When stress-induced despair was measured in the tail-suspension test, KIV mice showed significantly longer immobility time than the respective WT mice at all ages in both genders (Figure 1e, WT-SCT vs KIV-SCT, at least *P*<0.05). Collectively, these results indicated that promoter IV-BDNF deficiency reduced explorative activity in a novel environment and increased stress-induced despair, regardless of age and gender, whereas it caused age-dependent female-specific anxiety-like behavior.

### EET increased exploratory activity in both genotypes and normalized its reduction in KIV mice during ED and young adult stages, but not old adult

**Figure 2:**
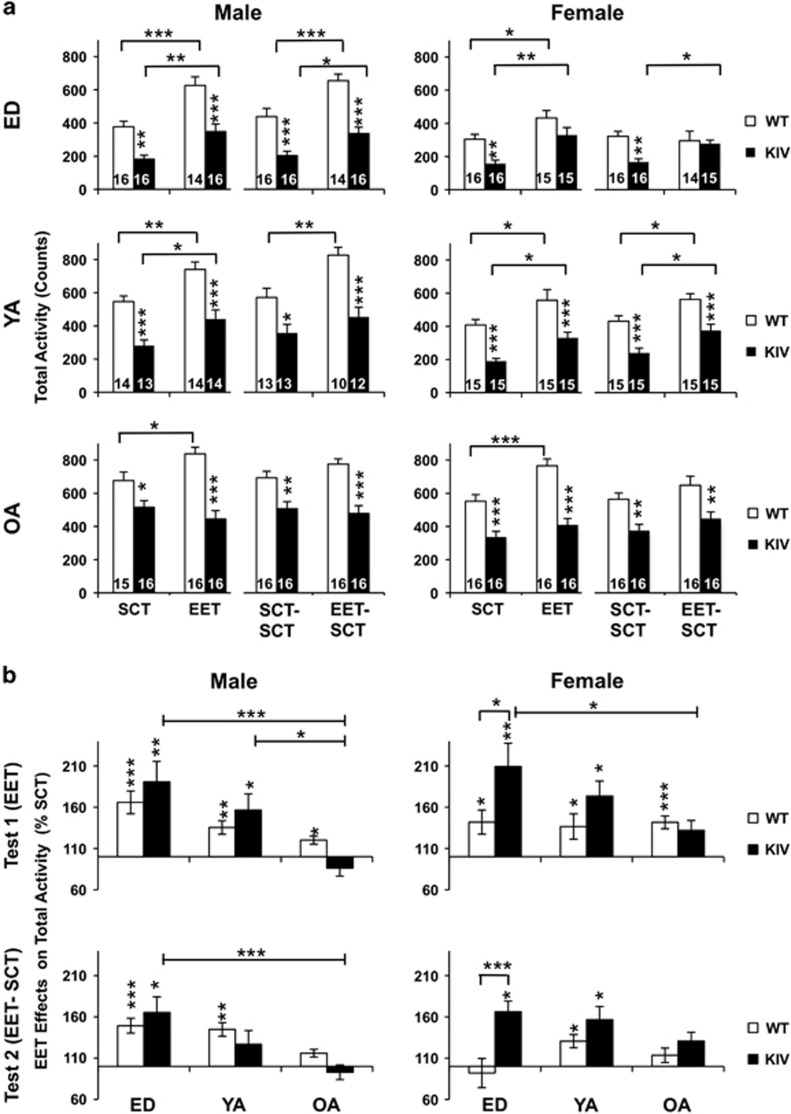
EET effects on total activity in the open-field test across ages (male: left; female: right). (**a**) Total activity in WT and KIV mice after 2 months of EET (SCT/EET, T_1_), then after 1 month of SCT (SCT–SCT/EET–SCT, T_2_). Asterisks on the columns show a significant difference between genotypes. (**b).** EET effects shown by % SCT across ages at T_1_ (top) and at T_2_ (bottom). Asterisks on the columns show a significant effect of EET compared with SCT. ED, early-life development; EET, enriched environment treatment; KIV, knock-in BDNF-promoter IV; OA, old adult; SCT, standard condition treatment; WT, wild-type; YA, young adult. *P<0.05, **P<0.01, ***P<0.005. *N=*10–16 (shown at the columns).

Next, we asked whether 2 months of EET during ED, young adult or old adult stages reversed the depression-like behavior of KIV mice. EET significantly increased total activity in the open-field test for all genotypes and genders (Figure 2a, SCT vs EET: at least *P*<0.05), except old adult KIV males and females (Figure 2a, *P*>0.05). Then, we determined whether EET effects on total activity persisted for 1 month without EET. Two-way analyses of variance with *post hoc* tests revealed that KIV mice with EET (EET–SCT), compared with KIV mice with SCT (SCT–SCT), showed significantly increased total activity in ED males and females and young adult females (Figure 2a, at least *P*<0.05), indicating sustained effects of EET. WT mice with EET (EET–SCT) also showed significantly increased total activity in ED males and young adult males and females, compared to WT mice with SCT (SCT–SCT, at least *P*<0.05). By contrast, no sustaining EET effects were observed in old adults of either genotype or gender (Figure 2a, bottom).

EET effects in increasing total activity were then compared across the three life stages. Analyses of variance revealed the strongest EET effects at ED, particularly in KIV mice of both genders (Figure 2b). Upon analyzing genotype effects, *post hoc* tests revealed larger EET effects in KIV females than in WT females at ED (*P*<0.05). The EET effects, although decreased (compare Figure 2b top vs bottom), lasted accordingly after 1 month of SCT (EET–SCT vs SCT–SCT: at least *P*<0.05 in ED and young adult groups, except ED WT females and young adult KIV males). Altogether, these results outlined the largest, long-lasting EET effects in increasing explorative activity at ED among the life stages, particularly in KIV mice.

When compared with EET effects on total activity, EET effects on distance moved in the open-field test were limited and dependent on genotype and gender. EET showed no effect on distance moved in KIV mice in either gender at any age, except that EET decreased it in old adult KIV males (Supplementary Figure 1a, KIV-EET vs KIV-SCT, *P*<0.01). In WT mice, EET increased distance moved in all age males (WT-EET vs WT-SCT, at least *P*<0.05) but decreased it in ED females (WT-EET vs WT-SCT, *P*<0.01). The EET effect did not last 1 month after EET discontinuance for any group, except that ED WT females showed a sustained reduction in distance moved (WT EET–SCT vs WT SCT–SCT, *P<*0.001). Accordingly, EET effects significantly differ between genotypes in old adult males and ED females (Supplementary Figure 1b, WT vs KIV at least *P<*0.05). Collectively, these results suggest that the increased total activity by EET does not always reflect the distance moved and that increased total activity by EET may be attributed to the explorative vertical activity (for example, rearing).

### EET increased anxiolytic behavior solely at ED in KIV mice, but at all stages in WT males and old adult WT females

**Figure 3:**
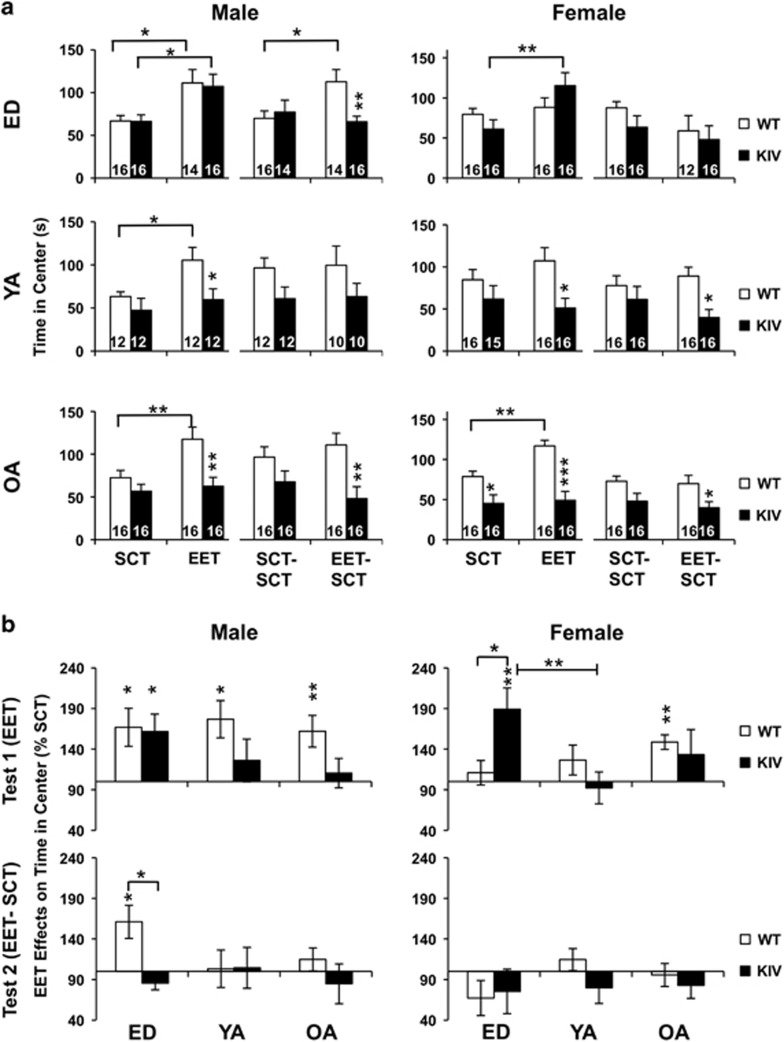
EET effects on time in the center in the open-field test across ages. (**a**) Time in center measured at T_1_ (SCT/EET) and at T_2_ (SCT–SCT/EET–SCT). Asterisks on the columns show a significant difference between genotypes. (**b**) EET effects shown as % SCT across ages at T_1_ (top) and at T_2_ (bottom). Asterisks on the columns show a significant effect of EET (vs SCT). ED, early-life development; EET, enriched environment treatment; OA, old adult; SCT, standard condition treatment; YA, young adult. *P<0.05, **P<0.01, ***P<0.005. *N=*10–16.

We also examined the anxiolytic effects of EET by measuring time spent in the center in the open-field test. EET increased time in the center in KIV mice only at ED for both genders but in all age WT males and old adult WT females (Figure 3a, at least *P<*0.05). The EET effects were largest at ED groups in KIV mice (Figure 3b, top). However, this EET effect only lasted in ED WT males after 1 month without EET (Figures 3a, *P<*0.05 and Figure 3b, bottom). These results indicated that anxiolytic effects of EET were largest at ED in promoter IV-BDNF deficiency, but this effect did not persist without EET.

### EET decreased stress-induced despair in both genotypes and normalized it in KIV mice during ED and young adult, but not old adult, stages

**Figure 4:**
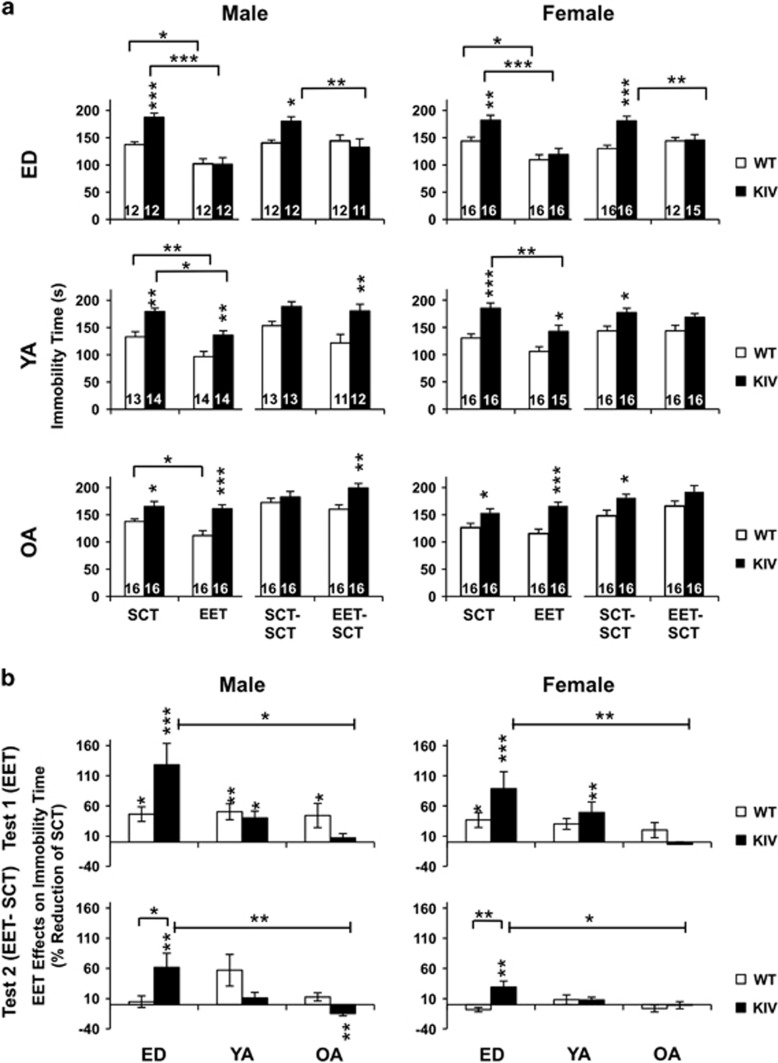
EET effects on immobility time in the tail-suspension test across ages. (**a**). Immobility time in WT and KIV mice at T_1_ (SCT/EET) and T_2_ (SCT–SCT/EET–SCT). Asterisks on the columns show a significant difference between genotypes. (**b**). EET effects shown as % SCT across ages at T_1_ (top) and at T_2_ (bottom). Asterisks on the columns show a significant effect of EET compared with SCT. ED, early-life development; EET, enriched environment treatment; KIV, knock-in BDNF-promoter IV; OA, old adult; SCT, standard condition treatment; WT, wild-type; YA, young adult. *P<0.05, **P<0.01, ***P<0.005. *N=*11–16.

We further determined EET effects on stress-induced despair using the tail-suspension test. EET significantly decreased immobility time in ED and young adult KIV mice (at least *P<*0.05), but not in old adult KIV mice, for both genders (Figure 4a). In WT mice, EET significantly decreased immobility time in all groups (at least *P<*0.05), except young and old adult females (Figure 4a). The EET effects were largest in ED KIV mice (Figure 4b, top) and lasted only in ED KIV mice for both genders with larger effects in KIV mice than WT mice (Figure 4b, bottom, *P<*0.05). These results indicated that, in promoter IV-BDNF deficiency, EET effects in decreasing stress-induced despair were largest and sustainable when EET was provided during ED, but this effect was limited when provided at old adulthood.

### EET, only at ED, restored BDNF levels in the hippocampus and frontal cortex of KIV mice; this effect persisted without EET only in the hippocampus of ED KIV mice

**Figure 5:**
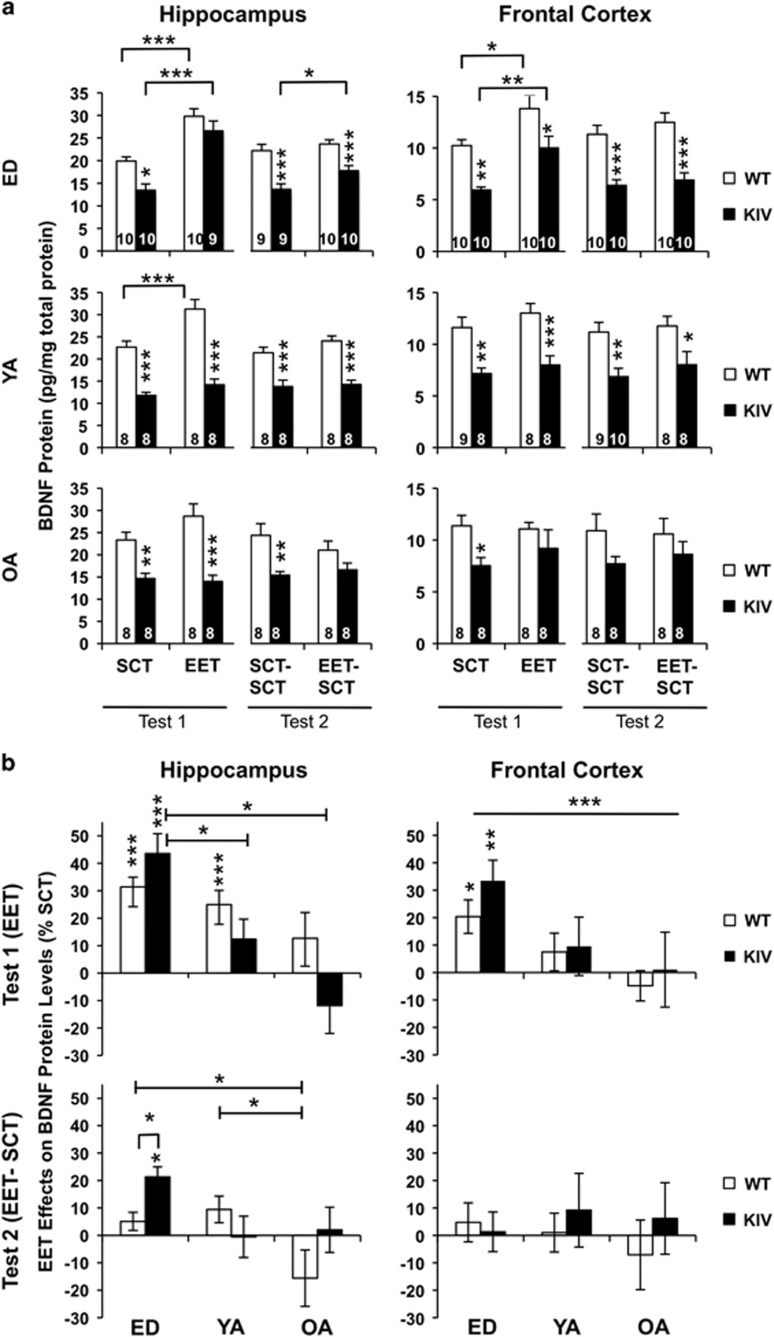
EET effects on BDNF protein levels in the hippocampus (left) and frontal cortex (right) measured by ELISA. (**a**) BDNF levels in WT and KIV mice after EET (SCT/EET, T_1_), then after 1 month of SCT (SCT–SCT/EET–SCT, T_2_). Asterisks on the columns show a significant difference between genotypes. Data are combined (*N=*8–10) from male (*N=*4–5) and female (*N=*4–5) mice because no gender differences were observed. (**b**) EET effects shown as % SCT across ages at T_1_ (top) and at T_2_ (bottom). Asterisks on the columns show a significant effect of EET compared to SCT. BDNF, brain-derived neurotrophic factor; ED, early-life development; EET, enriched environment treatment; KIV, knock-in BDNF-promoter IV; OA, old adult; SCT, standard condition treatment; WT, wild-type; YA, young adult. *P<0.05, **P<0.01, ***P<0.005.

Last, we asked whether the behavioral effects of EET reflect BDNF levels in the hippocampus and frontal cortex, two regions where dysfunction is related to depression. The data from males and females (*N=*4–5) were combined (total *N=*8–10, Figure 5) because no gender-specific differences were detected (*P*>0.05). With SCT, all KIV mice, when compared with WT mice, showed significantly reduced BDNF protein levels in the hippocampus and frontal cortex (Figure 5a, at least *P<*0.05). EET during ED significantly increased BDNF levels in these brain regions of both genotypes (SCT vs EET, at least *P<*0.05), normalizing its reduction in KIV mice (Figure 5a, WT-SCT vs KIV-EET: *P*>0.05). By contrast, EET at young or old adult stages did not increase BDNF levels in these brain regions in either genotype or gender (*P*>0.05), except in young adult WT males (Figure 5a, *P<*0.05). Thus, the EET effects in BDNF induction depended on age and were largest in ED groups in both WT and KIV mice (Figure 5b, hippocampus: F_2,45_=7.1, *P<*0.001; frontal cortex: F_2,46_=6.0, *P<*0.005). One month after EET discontinuance, the EET effects in BDNF induction lasted in the hippocampus of KIV mice (*P*<0.05, Figure 5a), an effect larger in KIV mice than in WT mice (Figure 5b, bottom). By contrast, the EET effects did not last in the frontal cortex in either genotype (Figures 5a and 5b).

## Discussion

Results of the present study demonstrated that (1) deficiency of promoter IV-driven BDNF reduces total activity in the open-field test and increases immobility time in the tail-suspension test, regardless of age and gender; (2) 2-month EET at ED, but not at old adulthood, normalized depression-like behavior caused by promoter IV-BDNF deficiency in KIV mice, with larger effects at ED than at young/old adulthood; (3) EET at ED, but not at old adulthood, increased and normalized BDNF protein levels in the hippocampus and frontal cortex of KIV mice, reflecting its antidepressive and anxiolytic effects; (4) 1 month after discontinuance of EET, the antidepressive and hippocampal BDNF effects of EET lasted in ED groups in promoter IV-BDNF deficiency, whereas anxiolytic and frontal cortex BDNF effects of EET did not; and (5) no gender-specific effects of EET were detected, except in the distance moved and time in center in the open-field test.

To our knowledge, this is the first study that shows EET effects on depressive behavior and BDNF levels across ages from early-life to old adulthood, in both normal and BDNF-deficient conditions. Many studies, including ours, have shown those effects of EET in a certain period of time or in normal conditons.^7, 28, 31, 32, 33, 48, 49, 50, 51^ Our results indicate that early-life EET is more effective than EET during young or old adulthood in decreasing depressive behavior and restoring BDNF levels, particularly in promoter IV-BDNF deficiency. Our findings supported our hypotheses, providing important insight into the lifetime when EET is most effective against depression caused by BDNF deficiency.

### Reduced BDNF levels and depression across ages

Our results suggest that promoter IV-BDNF deficiency, such as that occurring under chronic/traumatic stress and lack of activity,^20, 21, 23^ can cause depressive behavior, regardless of age and gender. In humans, depression is observed across all ages.^52^ Reduced brain BDNF levels and inactive promoter IV have been reported in depressed adult human patients,^19, 24, 25, 53, 54^ but remain unknown in younger patients. However, reduced blood BDNF levels have recently been reported in pediatric depression patients^53^ as well as in older depression patients.^54^ Blood BDNF levels have been reported to reflect brain BDNF levels in animals;^55^ thus, these reports implicate decreased brain BDNF levels in depression across ages.

Our results of depression-like behavior in KIV mice of both genders are in contrast to the previous studies showing unchanged depression-like behavior or female-specific depression-like behavior under stress in BDNF-coding region deficient mice (BDNF +/- and conditional knockouts, ^56, 57, 58, 59, 60^ reviews in Duman and Monteggia^13^ and Groves^61^). This may be because BDNF deficiency in the regions/cells that BDNF promoter IV controls (for example, dorsal regions, hippocampal CA1/prefrontal layer V cells), rather than in the whole brain (BDNF +/-) or in the conditional regions/cells controlled by exogenous promoters (for example, enolase, CaMKII and GFAP), leads to depression-like behavior (detailed discussions in Sakata *et al.*^10^). It is also possible that loss of BDNF in conditional knockouts is insufficient to cause depression-like behavior, but is prone to do so when BDNF levels are further reduced by inactive promoter IV under stress,^20, 21^ and females may be more susceptible to stress.

### Maximum antidepressive and BDNF effects of EET during ED

Early life is a time of dynamic neuronal development, shaped by environment and (epi)genetic controls.^40^ Our results showed that early-life EET was most effective in decreasing depression-like behavior and increasing BDNF levels in promoter IV-BDNF-deficient (KIV) mice. Antidepressive and hippocampal BDNF effects persisted for 1 month without EET, indicating that early-life EET may prevent depression later in life. The impact of early-life EET is substantial; it can break the vicious cycle of depressed mother and promoter IV-BDNF deficiency in infants.^21, 23^ Early-life maltreatment of infants has been shown to increase methylation of the promoter IV-controlled BDNF DNA in the prefrontal cortex and reduce BDNF expression from infancy to adulthood.^21^ Consequently, abusive maternal behavior and previously acquired DNA methylation patterns perpetually transmit from generation to generation.^21^ Inactivation of promoter IV can also occur after exposure to toxic environments: perinatal methylmercury exposure in mice leads to DNA hypermethylation, increased histone trimethylation, and decreased histone acetylation within promoter IV in the hippocampus of offspring and was associated with depression-like behavior.^62^ Early detection of BDNF deficiency (for example, measuring promoter IV inactivity and related biomarkers) may help in initiating early-life EET as intervention to reduce risk of depression later in life.

Our results showed that BDNF induction by EET was larger at ED than young or old adulthood and lasted without EET only in the hippocampus of ED KIV mice. We have previously shown that EET compensates for promoter IV-BDNF deficiency by activating other BDNF promoters.^7^ It is possible that lasting BDNF induction may arise from long-lasting epigenetic activation of the other promoters. Epigenetic regulations of BDNF promoters by early-life EET, in contrast to those caused by early-life stress, remain largely unknown. However, it is worth noting that communal nesting with maternal care-giving leads to increased histone acetylation at the BDNF promoter I and IV regions in the adult hippocampus, which is more permissive to expression.^63^ Pre-reproductive maternal enrichment has been also shown to increase frontal BDNF levels of mother mice, and their offspring express increased hippocampal BDNF levels.^64^ Exercise, an important element of EET that activates multiple promoters (I, II, III, IV and VI),^7, 31, 32, 33, 34, 35^ has been also shown to increase BDNF expression when provided earlier (that is, pregnancy, adolescence and young adulthood) rather than later in life.^32, 65, 66^ It remains to be addressed how long epigenetic regulations by early-life EET persist and whether they can be transgenerational.^67, 68^

In contrast to the well-known EET effects on BDNF levels in the hippocampus,^7, 28, 31, 32, 33, 48, 49, 50, 51^ effects in other regions are not established. Our results showed that BDNF was induced in the frontal cortex by EET only at ED, which did not persist. This suggests that there is a critical period for increasing BDNF levels in the frontal cortex to normalize its reduction due to promoter IV inactivation.^21^ A previous study has shown that treadmill exercise during pregnancy increased prefrontal cortex BDNF levels of rat pups at day 26 and at 4 months of age, while increasing locomotor activity and decreasing anxiety.^69^ However, similar to our results, pre-reproductive maternal enrichment increased BDNF levels in the hippocampus but not frontal cortex of offspring.^64, 68^ Our results also suggest that continuous EET may be needed to maintain BDNF induction in the frontal cortex. Future studies need to clarify when in ED (parental/gestation/before weaning/after weaning to adolescent/adolescent) EET is most effective for inducing BDNF expression and decreasing depressive behavior later in life, and which antidepressive effects and BDNF epigenetic conditions carry into adulthood and for how long.

Our results regarding anxiolytic effects of early-life EET are similar to a previous report.^70^ In contrast to the long-lasting antidepressive effects of early-life EET (Figures 2 and 4), its anxiolytic effects did not last after EET discontinuance (Figure 3). Continuous EET or much earlier EET may be needed for lasting anxiolytic effects; a study has shown prenatal EET (3-week gestation and 3-week postnatal) restored deficits in emotion-related behavior caused by adulthood stress in young adult rats.^71^ Further understanding early-life EET and mechanisms of epigenetic BDNF regulations^17, 72^ across ages may be valuable to reduce anxiety as well as other psychiatric disorders.^73, 74, 75^

### Mixed effects of EET during young adulthood

The presented data of EET effects in young adult mice mostly agree with our previous results^7, 10^ and others,^7, 28, 31, 32, 33, 48, 49, 50, 51^ except that our previous results showed hippocampal BDNF induction in young KIV adults, which was not observed in the current study. This discrepancy may be because EET length was longer (2 months) than previously (3 weeks); therefore, when EET effects were tested, the young adult mice in this study were slightly older (4–5 months vs ~3 months). EET effects may decline with age due to relatively fixed epigenetic regulation, and there may be a critical period between 3–4 months of age in mice to induce BDNF expression via other promoters. Previous studies have also shown mixed results in the induction of BDNF by EET, likely depending on when treatment begins and its length, as well as on gender. Previous studies have shown that hippocampal BDNF induction by EET (or exercise) is larger in males^76, 77^ or females,^78^ or similar between genders.^79^ The reasons of these mixed results are unknown, but could be because hippocampal BDNF levels are affected by estrous cycle and sex hormones.^80, 81, 82^ We did not observe any gender-specific differences in BDNF levels in either genotype or in BDNF induction by EET; the gender effect may be relatively smaller than the genotype effect (BDNF deficiency) and age-dependent environmental (EET) effect.

Our results showed that effects of EET during young adulthood, except for those increasing total activity, did not last 1 month after discontinuing EET, suggesting that constant EET is required after adulthood for antidepressive and BDNF effects. Another new finding of the present study is the dissociation between EET effects on the total activity and distance moved in the open-field test (compare Figure 2 and Supplementary Figure 1). EET increased total activity for both genotypes and genders, while EET increased distance moved in WT males but decreased it in WT females. This may explain the discrepancy in previous reports about EET effects on the activity in the open-field test: no effect^83, 84^ or reduction in distance moved.^28, 48, 49, 85^

### Limited effects of EET during old adulthood

Our results revealed limited EET effects in decreasing depression-like behavior or inducing BDNF levels in either the hippocampus or frontal cortex in old adult KIV mice. This suggests that depression caused by promoter IV-BDNF deficiency may not be reversed by EET after middle-age; other treatments (for example, antidepressants and electroconvulsive therapy) may be necessary.

Our results of no EET effects in old adult mice were somewhat unexpected because previous human studies have shown that aerobic training increases blood BDNF levels^86^ and that exercise, an important component of EET for BDNF induction,^28, 29^ correlates with^87^ and produces^88^ antidepressive effects in elderly depression patients. However, conflicting results have also been published: while increased exercise can decrease depression, already depressed individuals may not benefit from exercise.^89^ Moderately intense exercise has also failed to reduce human depressive symptoms.^90^ Similarly, old adult rodents have also showed mixed results in BDNF induction by EET or exercise, likely dependent upon treatment intensity and length. Short-term exercise (<3 weeks) increased,^32, 91^ but long-term exercise (4 weeks)^32^ did not increase hippocampal BDNF levels in 15–24-month old rodents. However, even longer exercise^6^ or EET^92^ (8–10 months) increased BDNF levels in the hippocampus and cerebral cortex in 9–15-month-old rodents. It is possible that longer, more intense EET regimens are needed to increase BDNF levels and depressive symptoms in aged subjects. Different EET components may also affect treatment efficacy at different ages: housing and social interaction rather than physical exercise could be more effective for old animals than younger animals.^93^

One caveat in the present study is that animals' responsiveness to EET was voluntary and uncontrolled. It is possible that old adult mice reacted to the enriched environment much less than younger mice (for example, less running and responses to new objects/other mice), which limited antidepressive and BDNF effects. Indeed, middle-aged (15 months) and older (24 months) adult mice show reduced voluntary exercise than younger (2 months) adult mice.^32^ Forced behavioral changes (for example, exercise on a treadmill) may yield EET effects in aged adults but may also cause stress. Future studies measuring animal responsivity to EET—amounts of physical exercise (for example, running length), mental exercise (for example, contact time with toys), and social interactions (for example, contact time with other mice)—may clarify the animals' responsiveness to the EET and identify the critical components of EET (physical, mental, or social activity) that are responsible for achieving antidepressive effects and BDNF induction. It should also be noted that many other molecules/systems (for example, other neurotrophines, monoamines, and neurogenesis)^3, 38, 92^ may also mediate the antidepressive effects^94, 95^ in BDNF-deficient conditions across ages, which requires further examination.

## Conclusion

The present study found that EET during ED was more effective than EET during young or old adulthood in decreasing depressive behaviors and increasing BDNF levels, regardless of gender in both normal and promoter IV-BDNF-deficient conditions. The effects induced by early-life EET lasted after its discontinuance, particularly in promoter IV-BDNF deficiency, indicating long-lasting compensational effects of early-life EET. Our results suggest that EET provided during early development is particularly beneficial to individuals with depression caused by promoter IV-BDNF deficiency, while additional treatment may be needed for older adults. Future studies are needed to determine the precise critical period of maximal EET effects and its detailed underlying mechanisms. This will help in understanding how lifetime and epigenetic predisposition interfere with EET's antidepressive effects and provide strategies for developing a more effective prevention and treatment for depression.
