# Supplementary material for: Antidepressive and BDNF effects of enriched environment treatment across ages in mice lacking BDNF expression through promoter IV
Source: Transl Psychiatry. 2016 Sep 20;6(9):e896–. doi: 10.1038/tp.2016.160 (PMC5048201; doi:10.1038/tp.2016.160)
Supplement: Supplementary Information [file tp2016160x1.pdf]

Supplementary Information

1. Supplementary Table 1.

2. Supplementary Fig. 1

3. Figure legends for supplementary Fig. 1

Supplementary Fig. 1. Effects of enriched environment treatment (EET) on distance moved in the open field test across ages (male: left; female: right). a. Distance moved in WT and KIV mice after 2 months of EET (SCT and EET, T_1), then after 1 month of SCT (SCT-SCT and EET-SCT, T_2). Asterisks on the columns show a significant difference between WT and KIV mice. **b.** EET effects shown as % SCT across ages at T_1 (top) and at T_2 (bottom). Asterisks on the columns show a significant effect of EET compared to SCT. ED, early-life development; YA, young adult; OA, old adult. *P<0.05, **P<0.01, ***P<0.005. N=10–16 (shown at the columns).
