# Supplementary material for: Antidepressive and BDNF effects of enriched environment treatment across ages in mice lacking BDNF expression through promoter IV
Source: Transl Psychiatry. 2016 Sep 20;6(9):e896–. doi: 10.1038/tp.2016.160 (PMC5048201; doi:10.1038/tp.2016.160)
Supplement: Supplementary Table 1 [file tp2016160x2.pdf]

Supplementary Table 1. Statistical Analysis

1. Two-way ANOVA: Genotype and age effects.

**Fig. 1b-e Effects of promoter IV-BDNF deficiency across ages**			**Genotype Effects**	**Age Effects**	**Genotype X Age Interactions**	***Test 2 hoc* Bonferroni Test Genotype Effects**			***Test 2 hoc* Bonferroni Test Aging Effects**		
						**ED**	**YA**	**OA**	**ED vs. YA**	**ED vs. OA**	**YA vs. OA**
Male	OFT	Total Activity	**$F_{(1,84)} =$ 54.9 $P < 0.001$**	$F_{(2,84)} =$ 2.6 $P = 0.08$	$F_{(2,84)} =$ 2.6 $P = 0.08$	**$T_{30} = 5.5$ $P < 0.001$**	**$T_{25} = 4.7$ $P < 0.001$**	**$T_{29} = 2.5$ $P < 0.05$**	$T_{27} = 5.5$ $P > 0.05$	**$T_{30} = 3.0$ $P < 0.05$**	$T_{30} = 2.5$ $P > 0.05$
Male	OFT	Distance Moved	$F_{(1,86)} =$ 3.7 $P = 0.06$	$F_{(2,86)} =$ 0.25 $P = 0.78$	$F_{(2,86)} =$ 0.25 $P = 0.78$	$T_{30} = 1.3$ $P > 0.05$	$T_{26} = 1.5$ $P > 0.05$	$T_{30} = 0.6$ $P > 0.05$	$T_{28} = 0.3$ $P > 0.05$	$T_{30} = 0.7$ $P > 0.05$	$T_{30} = 1.0$ $P > 0.05$
Male	OFT	Time in Center	$F_{(1,82)} =$ 3.3 $P = 0.07$	$F_{(2,82)} =$ 0.90 $P = 0.41$	$F_{(2,82)} =$ 0.93 $P = 0.40$	$T_{30} =$ 0.02 $P > 0.05$	$T_{25} = 1.8$ $P > 0.05$	$T_{29} = 1.3$ $P > 0.05$	$T_{26} = 1.9$ $P > 0.05$	$T_{30} = 1.2$ $P > 0.05$	$T_{30} = 0.73$ $P > 0.05$
Male	TST	Immobility Time	**$F_{(1,77)} =$ 39.0 $P < 0.001$**	$F_{(2,77)} =$ 1.1 $P = 0.32$	$F_{(2,77)} =$ 1.2 $P = 0.32$	**$T_{30} = 4.4$ $P < 0.001$**	**$T_{25} = 3.5$ $P < 0.01$**	**$T_{29} = 2.8$ $P < 0.05$**	$T_{24} = 1.2$ $P > 0.05$	$T_{26} = 2.1$ $P > 0.05$	$T_{26} = 0.92$ $P > 0.05$
Female	OFT	Total Activity	**$F_{(1,86)} =$ 70.8 $P < 0.001$**	$F_{(2,86)} =$ 0.52 $P = 0.59$	$F_{(2,86)} =$ 0.50 $P = 0.61$	**$T_{30} = 5.1$ $P < 0.001$**	**$T_{28} = 5.4$ $P < 0.001$**	**$T_{28} = 4.0$ $P < 0.001$**	$T_{29} = 0.48$ $P > 0.05$	$T_{29} = 0.54$ $P > 0.05$	$T_{29} = 1.0$ $P > 0.05$
Female	OFT	Distance Moved	**$F_{(1,90)} =$ 29.8 $P < 0.001$**	$F_{(2,90)} =$ 1.1 $P = 0.35$	$F_{(2,90)} =$ 1.1 $P = 0.35$	**$T_{30} = 4.3$ $P < 0.001$**	$T_{30} = 2.4$ $P > 0.05$	**$T_{30} = 2.8$ $P < 0.05$**	$T_{30} = 2.0$ $P > 0.05$	$T_{30} = 1.5$ $P > 0.05$	$T_{30} = 0.41$ $P > 0.05$
Female	OFT	Time in Center	**$F_{(1,88)} =$ 11.5 $P < 0.001$**	$F_{(2,88)} =$ 0.76 $P = 0.47$	$F_{(2,88)} =$ 0.76 $P = 0.47$	$T_{30} = 1.7$ $P > 0.05$	$T_{25} = 1.2$ $P > 0.05$	**$T_{29} = 3.0$ $P < 0.05$**	$T_{28} = 0.39$ $P > 0.05$	$T_{29} = 1.3$ $P > 0.05$	$T_{29} = 1.6$ $P > 0.05$
Female	TST	Immobility Time	**$F_{(1,89)} =$ 37.1 $P < 0.001$**	$F_{(2,89)} =$ 1.2 $P = 0.30$	$F_{(2,89)} =$ 1.2 $P = 0.30$	**$T_{30} = 3.0$ $P < 0.01$**	**$T_{25} = 4.8$ $P < 0.001$**	**$T_{29} = 2.7$ $P < 0.05$**	$T_{30} = 1.8$ $P > 0.05$	$T_{29} = 0.28$ $P > 0.05$	$T_{29} = 2.0$ $P > 0.05$

2. Two-way ANOVA: Genotype and EET effects.

Fig. 2a<br>OFT<br>Total Activity			Genotype Effects	Treatment Effects	Genotype X Treatment Interactions	*Test 2 hoc* Bonferroni Test Genotype Effects (WT vs KIV)		*Test 2 hoc* Bonferroni Test Treatment Effects (SCT vs EET)	
						SCT	EET	WT	KIV
Male	Test 1 (T_1)	ED	**$F_{(1,58)} = 36.4$<br>$P < 0.0001$**	**$F_{(1,58)} = 28.0$<br>$P < 0.0001$**	$F_{(1,58)} = 1.1$<br>$P = 0.29$	**$T_{30} = 3.6$<br>$P < 0.01$**	**$T_{28} = 4.9$<br>$P < 0.0001$**	**$T_{28} = 4.4$<br>$P < 0.0001$**	**$T_{30} = 3.0$<br>$P < 0.01$**
		YA	**$F_{(1,51)} = 42.8$<br>$P < 0.0001$**	**$F_{(1,51)} = 16.8$<br>$P < 0.0001$**	$F_{(1,51)} = 0.16$<br>$P = 0.69$	**$T_{25} = 4.3$<br>$P < 0.001$**	**$T_{26} = 5.0$<br>$P < 0.0001$**	**$T_{26} = 3.2$<br>$P < 0.01$**	**$T_{25} = 2.6$<br>$P < 0.05$**
		OA	**$F_{(1,59)} = 38.9$<br>$P < 0.0001$**	$F_{(1,59)} = 1.1$<br>$P = 0.431$	**$F_{(1,59)} = 6.8$<br>$P < 0.05$**	**$T_{29} = 2.5$<br>$P < 0.05$**	**$T_{30} = 6.3$<br>$P < 0.0001$**	**$T_{29} = 2.6$<br>$P < 0.05$**	$T_{30} = 1.1$<br>$P > 0.05$
	Test 2 (T_2)	ED	**$F_{(1,58)} = 49.1$<br>$P < 0.0001$**	**$F_{(1,58)} = 19.5$<br>$P < 0.0001$**	$F_{(1,58)} = 1.1$<br>$P = 0.30$	**$T_{30} = 4.3$<br>$P < 0.001$**	**$T_{28} = 5.6$<br>$P < 0.0001$**	**$T_{28} = 3.8$<br>$P < 0.001$**	**$T_{30} = 2.4$<br>$P < 0.05$**
		YA	**$F_{(1,44)} = 28.7$<br>$P < 0.0001$**	**$F_{(1,44)} = 10.3$<br>$P < 0.005$**	$F_{(1,44)} = 2.1$<br>$P = 0.15$	**$T_{24} = 2.9$<br>$P < 0.05$**	**$T_{20} = 4.6$<br>$P < 0.0001$**	**$T_{21} = 3.2$<br>$P < 0.01$**	$T_{23} = 1.3$<br>$P > 0.05$
		OA	**$F_{(1,60)} = 40.3$<br>$P < 0.0001$**	$F_{(1,60)} = 0.48$<br>$P = 0.49$	**$F_{(1,60)} = 5.0$<br>$P < 0.05$**	**$T_{30} = 3.3$<br>$P < 0.01$**	**$T_{30} = 5.2$<br>$P < 0.0001$**	$T_{30} = 1.4$<br>$P > 0.05$	$T_{30} = 0.5$<br>$P > 0.05$
Female	Test 1 (T_1)	ED	**$F_{(1,58)} = 12.4$<br>$P < 0.001$**	**$F_{(1,58)} = 17.9$<br>$P < 0.0001$**	$F_{(1,58)} = 0.4$<br>$P = 0.53$	**$T_{30} = 3.0$<br>$P < 0.01$**	$T_{28} = 2.0$<br>$P > 0.05$	**$T_{29} = 2.5$<br>$P < 0.05$**	**$T_{29} = 3.4$<br>$P < 0.01$**
		YA	**$F_{(1,56)} = 32.4$<br>$P < 0.0001$**	**$F_{(1,56)} = 13.9$<br>$P < 0.0005$**	$F_{(1,56)} = 0.01$<br>$P = 0.91$	**$T_{28} = 4.2$<br>$P < 0.001$**	**$T_{28} = 4.8$<br>$P < 0.0001$**	**$T_{28} = 2.7$<br>$P < 0.05$**	**$T_{28} = 2.6$<br>$P < 0.05$**
		OA	**$F_{(1,60)} = 55.1$<br>$P < 0.0001$**	**$F_{(1,60)} = 14.0$<br>$P < 0.001$**	**$F_{(1,60)} = 3.4$<br>$P = 0.07$**	**$T_{30} = 4.0$<br>$P < 0.001$**	**$T_{30} = 6.5$<br>$P < 0.0001$**	**$T_{30} = 3.9$<br>$P < 0.001$**	$T_{30} = 1.4$<br>$P > 0.05$
	Test 2 (T_2)	ED	**$F_{(1,57)} = 6.5$<br>$P < 0.05$**	$F_{(1,57)} = 1.6$<br>$P = 0.22$	**$F_{(1,57)} = 4.1$<br>$P < 0.05$**	**$T_{30} = 3.3$<br>$P < 0.01$**	$T_{27} = 0.4$<br>$P > 0.05$	$T_{28} = 0.5$<br>$P > 0.05$	**$T_{29} = 2.3$<br>$P < 0.05$**
		YA	**$F_{(1,56)} = 30.8$<br>$P < 0.0001$**	**$F_{(1,56)} = 15.3$<br>$P < 0.001$**	$F_{(1,56)} < 0.01$<br>$P = 0.96$	**$T_{28} = 4.0$<br>$P < 0.001$**	**$T_{28} = 3.9$<br>$P < 0.001$**	**$T_{28} = 2.7$<br>$P < 0.05$**	**$T_{28} = 2.8$<br>$P < 0.05$**
		OA	**$F_{(1,60)} = 19.9$<br>$P < 0.0001$**	$F_{(1,60)} = 3.3$<br>$P = 0.08$	$F_{(1,60)} = 0.02$<br>$P = 0.90$	**$T_{30} = 3.1$<br>$P < 0.01$**	**$T_{30} = 3.2$<br>$P < 0.01$**	$T_{30} = 1.4$<br>$P > 0.05$	$T_{30} = 1.2$<br>$P > 0.05$

Fig. 3a<br>OFT<br>Time in Center			Genotype Effects	Treatment Effects	Genotype X Treatment Interactions	*Test 2 hoc* Bonferroni Test Genotype Effects (WT vs KIV)		*Test 2 hoc* Bonferroni Test Treatment Effects (SCT vs EET)	
						SCT	EET	WT	KIV
Male	Test 1 (T_1)	ED	$F_{(1,58)} = 0.04$<br>$P = 0.83$	**$F_{(1,58)} = 13.9$<br>$P < 0.001$**	$F_{(1,58)} = 0.03$<br>$P = 0.87$	$T_{30} = 0.03$<br>$P > 0.05$	$T_{28} = 0.26$<br>$P > 0.05$	**$T_{28} = 2.7$<br>$P < 0.05$**	**$T_{30} = 2.6$<br>$P < 0.05$**
		YA	**$F_{(1,44)} = 6.4$<br>$P < 0.05$**	**$F_{(1,44)} = 5.0$<br>$P < 0.05$**	$F_{(1,44)} = 1.5$<br>$P = 0.22$	$T_{22} = 0.93$<br>$P > 0.05$	**$T_{22} = 2.7$<br>$P < 0.05$**	**$T_{22} = 2.5$<br>$P < 0.05$**	$T_{22} = 0.72$<br>$P > 0.05$
		OA	**$F_{(1,60)} = 11.1$<br>$P < 0.001$**	**$F_{(1,60)} = 5.8$<br>$P < 0.01$**	$F_{(1,60)} = 3.4$<br>$P = 0.070$	$T_{30} = 1.1$<br>$P > 0.05$	**$T_{30} = 3.7$<br>$P < 0.01$**	**$T_{30} = 3.0$<br>$P < 0.01$**	$T_{30} = 0.39$<br>$P > 0.05$
	Test 2 (T_2)	ED	$F_{(1,56)} = 3.2$<br>$P = 0.08$	$F_{(1,56)} = 2.1$<br>$P = 0.16$	**$F_{(1,56)} = 6.0$<br>$P < 0.05$**	$T_{28} = 0.5$<br>$P > 0.05$	**$T_{28} = 3.0$<br>$P < 0.01$**	**$T_{28} = 2.7$<br>$P < 0.05$**	$T_{28} = 0.7$<br>$P > 0.05$
		YA	**$F_{(1,44)} = 5.2$<br>$P < 0.05$**	$F_{(1,40)} = 0.04$<br>$P = 0.85$	$F_{(1,40)} = 0.01$<br>$P = 0.99$	$T_{22} = 1.7$<br>$P > 0.05$	$T_{18} = 1.6$<br>$P > 0.05$	$T_{20} = 0.15$<br>$P > 0.05$	$T_{20} = 0.13$<br>$P > 0.05$
		OA	**$F_{(1,60)} = 10.1$<br>$P < 0.01$**	$F_{(1,60)} = 0.20$<br>$P = 0.65$	$F_{(1,60)} = 1.0$<br>$P = 0.32$	$T_{30} = 1.6$<br>$P > 0.05$	**$T_{29} = 3.4$<br>$P < 0.01$**	$T_{29} = 0.39$<br>$P > 0.05$	$T_{30} = 1.0$<br>$P > 0.05$
Female	Test 1 (T_1)	ED	$F_{(1,60)} < 0.01$<br>$P = 0.95$	**$F_{(1,60)} = 5.3$<br>$P < 0.05$**	**$F_{(1,60)} = 4.7$<br>$P < 0.05$**	$T_{30} = 1.5$<br>$P > 0.05$	$T_{30} = 1.6$<br>$P > 0.05$	$T_{30} = 0.1$<br>$P > 0.05$	**$T_{30} = 3.2$<br>$P < 0.01$**
		YA	$F_{(1,60)} = 3.2$<br>$P = 0.08$	$F_{(1,60)} = 0.08$<br>$P = 0.78$	$F_{(1,60)} = 2.6$<br>$P = 0.11$	$T_{30} = 0.12$<br>$P > 0.05$	**$T_{30} = 2.4$<br>$P < 0.05$**	$T_{30} = 0.94$<br>$P > 0.05$	$T_{30} = 1.3$<br>$P > 0.05$
		OA	**$F_{(1,60)} = 30.2$<br>$P < 0.0001$**	**$F_{(1,60)} = 5.2$<br>$P < 0.05$**	$F_{(1,60)} = 3.6$<br>$P = 0.065$	**$T_{30} = 2.6$<br>$P < 0.05$**	**$T_{30} = 5.2$<br>$P < 0.0001$**	**$T_{30} = 2.9$<br>$P < 0.01$**	$T_{30} = 0.28$<br>$P > 0.05$
	Test 2 (T_2)	ED	$F_{(1,56)} = 1.4$<br>$P = 0.24$	$F_{(1,56)} = 2.2$<br>$P = 0.14$	$F_{(1,56)} = 0.19$<br>$P = 0.66$	$T_{30} = 1.2$<br>$P > 0.05$	$T_{26} = 0.5$<br>$P > 0.05$	$T_{26} = 1.3$<br>$P > 0.05$	$T_{30} = 0.8$<br>$P > 0.05$
		YA	**$F_{(1,60)} = 7.5$<br>$P < 0.01$**	$F_{(1,60)} = 0.18$<br>$P = 0.68$	$F_{(1,60)} = 1.9$<br>$P = 0.18$	$T_{30} = 0.97$<br>$P > 0.05$	**$T_{30} = 2.9$<br>$P < 0.05$**	$T_{30} = 0.67$<br>$P > 0.05$	$T_{30} = 1.3$<br>$P > 0.05$
		OA	**$F_{(1,60)} = 9.9$<br>$P < 0.005$**	$F_{(1,60)} = 0.43$<br>$P = 0.52$	$F_{(1,60)} = 0.09$<br>$P = 0.77$	$T_{30} = 2.0$<br>$P > 0.05$	**$T_{30} = 2.4$<br>$P < 0.05$**	$T_{30} = 0.25$<br>$P > 0.05$	$T_{30} = 0.67$<br>$P > 0.05$

Fig. 4a TST Immobility Time			Genotype Effects	Treatment Effects	Genotype X Treatment Interactions	*Test 2 hoc* Bonferroni Test Genotype Effects (WT vs KIV)		*Test 2 hoc* Bonferroni Test Treatment Effects (SCT vs EET)	
						SCT	EET	WT	KIV
Male	Test 1 (T_1)	ED	**$F_{(1,44)} = 7.6$ P < 0.01**	**$F_{(1,44)} = 46.1$ P < 0.0001**	**$F_{(1,44)} = 8.1$ P < 0.01**	**$T_{22} = 4.0$ P < 0.001**	$T_{22} = 0.1$ P > 0.05	**$T_{22} = 2.8$ P < 0.05**	**$T_{22} = 6.8$ P < 0.0001**
		YA	**$F_{(1,49)} = 21.2$ P < 0.0001**	**$F_{(1,49)} = 17.5$ P < 0.0001**	$F_{(1,49)} = 0.01$ P = 0.91	**$T_{25} = 3.1$ P < 0.01**	**$T_{26} = 3.3$ P < 0.01**	**$T_{25} = 3.1$ P < 0.01**	**$T_{26} = 2.9$ P < 0.05**
		OA	**$F_{(1,60)} = 24.1$ P < 0.0001**	$F_{(1,60)} = 3.9$ P = 0.05	$F_{(1,60)} = 2.0$ P = 0.17	**$T_{30} = 2.5$ P < 0.05**	**$T_{30} = 4.5$ P < 0.0001**	**$T_{30} = 2.4$ P < 0.05**	$T_{30} = 0.41$ P > 0.05
	Test 2 (T_2)	ED	$F_{(1,43)} = 1.9$ P = 0.18	**$F_{(1,43)} = 4.4$ P < 0.05**	**$F_{(1,43)} = 6.2$ P < 0.05**	**$T_{22} = 2.8$ P < 0.05**	$T_{21} = 0.8$ P > 0.05	$T_{22} = 0.3$ P > 0.05	**$T_{21} = 3.2$ P < 0.01**
		YA	**$F_{(1,54)} = 15.6$ P = 0.0002**	$F_{(1,54)} = 0.51$ P = 0.48	$F_{(1,54)} = 0.06$ P = 0.80	$T_{24} = 2.3$ P > 0.05	**$T_{21} = 3.6$ P < 0.01**	$T_{22} = 0.72$ P > 0.05	$T_{23} = 0.31$ P > 0.05
		OA	**$F_{(1,60)} = 7.9$ P < 0.01**	$F_{(1,60)} = 0.05$ P = 0.82	$F_{(1,60)} = 2.8$ P = 0.10	$T_{30} = 0.81$ P > 0.05	**$T_{30} = 3.1$ P < 0.01**	$T_{30} = 1.0$ P > 0.05	$T_{30} = 1.3$ P > 0.05
Female	Test 1 (T_1)	ED	**$F_{(1,60)} = 6.8$ P < 0.05**	**$F_{(1,60)} = 27.6$ P < 0.0001**	$F_{(1,60)} = 2.4$ P = 0.12	**$T_{30} = 2.9$ P < 0.01**	$T_{30} = 0.7$ P > 0.05	**$T_{30} = 2.6$ P < 0.05**	**$T_{30} = 4.8$ P < 0.0001**
		YA	**$F_{(1,60)} = 21.3$ P < 0.0001**	**$F_{(1,60)} = 14.9$ P < 0.001**	$F_{(1,60)} = 0.49$ P = 0.49	**$T_{30} = 3.8$ P < 0.001**	**$T_{29} = 2.8$ P < 0.05**	$T_{30} = 2.2$ P > 0.05	**$T_{29} = 3.2$ P < 0.01**
		OA	**$F_{(1,60)} = 26.5$ P < 0.0001**	$F_{(1,60)} = 0.02$ P = 0.89	$F_{(1,60)} = 2.5$ P = 0.12	**$T_{30} = 2.5$ P < 0.05**	**$T_{30} = 4.8$ P < 0.0001**	$T_{30} = 1.0$ P > 0.05	$T_{30} = 1.2$ P > 0.05
	Test 2 (T_2)	ED	**$F_{(1,55)} = 10.3$ P < 0.005**	$F_{(1,55)} = 1.7$ P = 0.20	**$F_{(1,55)} = 9.3$ P < 0.005**	**$T_{30} = 4.6$ P < 0.0001**	$T_{25} = 0.1$ P > 0.05	$T_{26} = 1.2$ P > 0.05	**$T_{29} = 3.2$ P < 0.01**
		YA	**$F_{(1,60)} = 11.7$ P < 0.005**	$F_{(1,60)} = 0.26$ P = 0.61	$F_{(1,60)} = 0.25$ P = 0.62	**$T_{30} = 2.8$ P < 0.05**	$T_{30} = 2.1$ P > 0.05	$T_{30} = 0.01$ P > 0.05	$T_{30} = 0.71$ P > 0.05
		OA	**$F_{(1,60)} = 9.5$ P < 0.005**	$F_{(1,60)} = 2.3$ P = 0.13	$F_{(1,60)} = 0.13$ P = 0.72	**$T_{30} = 2.4$ P < 0.05**	$T_{30} = 1.9$ P > 0.05	$T_{30} = 1.3$ P > 0.05	$T_{30} = 0.83$ P > 0.05

Fig. 5a BDNF ELISA (Males + Females)			Genotype Effects	Treatment Effects	Genotype X Treatment Interactions	*Test 2 hoc* Bonferroni Test Genotype Effects (WT vs KIV)		*Test 2 hoc* Bonferroni Test Treatment Effects (SCT vs EET)	
						SCT	EET	WT	KIV
Hippocampus	Test 1 (T_1)	ED	**$F_{(1,35)} = 9.0$ P < 0.005**	**$F_{(1,35)} = 50.6$ P < 0.0001**	$F_{(1,35)} = 0.90$ P = 0.35	**$T_{18} = 2.8$ P < 0.05**	$T_{17} = 1.4$ P > 0.05	**$T_{18} = 4.4$ P < 0.001**	**$T_{17} = 5.6$ P < 0.0001**
		YA	**$F_{(1,28)} = 90.5$ P < 0.0001**	**$F_{(1,28)} = 14.9$ P < 0.001**	**$F_{(1,28)} = 4.5$ P < 0.05**	**$T_{14} = 5.22$ P < 0.0001**	**$T_{14} = 8.23$ P < 0.0001**	**$T_{14} = 4.2$ P < 0.001**	$T_{14} = 1.2$ P > 0.05
		OA	**$F_{(1,28)} = 39.4$ P < 0.0001**	$F_{(1,28)} = 1.6$ P = 0.21	$F_{(1,28)} = 3.0$ P = 0.094	**$T_{14} = 3.2$ P < 0.01**	**$T_{14} = 5.7$ P < 0.0001**	$T_{14} = 2.1$ P > 0.05	$T_{14} = 0.33$ P > 0.05
	Test 2 (T_2)	ED	**$F_{(1,34)} = 50.3$ P < 0.0001**	**$F_{(1,34)} = 7.9$ P < 0.01**	$F_{(1,34)} = 1.9$ P = 0.18	**$T_{16} = 5.83$ P < 0.0001**	**$T_{18} = 4.15$ P < 0.001**	$T_{17} = 1.02$ P > 0.05	**$T_{17} = 2.96$ P < 0.05**
		YA	**$F_{(1,30)} = 51.4$ P < 0.0001**	$F_{(1,30)} = 0.55$ P = 0.47	$F_{(1,30)} = 1.8$ P = 0.19	**$T_{14} = 4.1$ P < 0.001**	**$T_{14} = 6.0$ P < 0.0001**	$T_{14} = 1.5$ P > 0.05	$T_{14} = 0.42$ P > 0.05
		OA	**$F_{(1,28)} = 12.4$ P < 0.005**	$F_{(1,28)} = 0.35$ P = 0.56	$F_{(1,28)} = 1.7$ P = 0.20	**$T_{14} = 3.4$ P < 0.01**	$T_{14} = 1.6$ P > 0.05	$T_{14} = 1.3$ P > 0.05	$T_{14} = 0.51$ P > 0.05
Frontal Cortex	Test 1 (T_1)	ED	**$F_{(1,36)} = 17.3$ P < 0.005**	**$F_{(1,36)} = 16.0$ P < 0.0005**	$F_{(1,36)} = 0.09$ P = 0.77	**$T_{18} = 3.2$ P < 0.01**	**$T_{18} = 2.7$ P < 0.05**	**$T_{18} = 2.6$ P < 0.05**	**$T_{18} = 3.0$ P < 0.01**
		YA	**$F_{(1,29)} = 27.4$ P < 0.0001**	$F_{(1,29)} = 2.1$ P = 0.16	$F_{(1,29)} = 0.20$ P = 0.66	**$T_{15} = 3.4$ P < 0.01**	**$T_{14} = 4.0$ P < 0.001**	$T_{15} = 1.4$ P > 0.05	$T_{14} = 0.70$ P > 0.05
		OA	**$F_{(1,28)} = 6.3$ P < 0.05**	$F_{(1,28)} = 0.35$ P = 0.56	$F_{(1,28)} = 0.73$ P = 0.40	**$T_{14} = 2.4$ P < 0.05**	$T_{14} = 1.2$ P > 0.05	$T_{14} = 0.19$ P > 0.05	$T_{14} = 1.0$ P > 0.05
	Test 2 (T_2)	ED	**$F_{(1,36)} = 41.5$ P < 0.0001**	$F_{(1,36)} = 0.90$ P = 0.35	$F_{(1,36)} = 0.21$ P = 0.65	**$T_{18} = 4.2$ P < 0.001**	**$T_{18} = 4.9$ P < 0.0001**	$T_{18} = 1.00$ P > 0.05	$T_{18} = 0.34$ P > 0.05
		YA	**$F_{(1,29)} = 15.6$ P < 0.001**	$F_{(1,29)} = 0.58$ P = 0.45	$F_{(1,29)} = 0.13$ P = 0.72	**$T_{15} = 3.1$ P < 0.01**	**$T_{14} = 2.5$ P < 0.05**	$T_{15} = 0.29$ P > 0.05	$T_{14} = 0.78$ P > 0.05
		OA	$F_{(1,28)} = 3.9$ P = 0.06	$F_{(1,28)} = 0.06$ P = 0.81	$F_{(1,28)} = 0.23$ P = 0.64	$T_{14} = 1.7$ P > 0.05	$T_{14} = 1.1$ P > 0.05	$T_{14} = 0.17$ P > 0.05	$T_{14} = 0.56$ P > 0.05

**Suppl. Fig. 1 OFT (Distance Moved)**			**Genotype Effects**	**Treatment Effects**	**Genotype X Treatment Interactions**	***Test 2 hoc* Bonferroni Test Genotype Effects (WT vs KIV)**		***Test 2 hoc* Bonferroni Test Treatment Effects (SCT vs EET)**	
						**SCT**	**EET**	**WT**	**KIV**
Male	Test 1 (T_1)	ED	**$F_{(1,57)} = 5.9$**<br>**P < 0.05**	**$F_{(1,57)} = 6.4$**<br>**P < 0.05**	$F_{(1,57)} = 0.8$<br>P = 0.37	$T_{30} = 1.1$<br>P > 0.05	$T_{27} = 2.3$<br>P > 0.05	**$T_{28} = 2.4$**<br>**P < 0.05**	$T_{29} = 1.2$<br>P > 0.05
		YA	**$F_{(1,52)} = 12.3$**<br>**P < 0.001**	**$F_{(1,52)} = 4.3$**<br>**P < 0.05**	$F_{(1,52)} = 2.4$<br>P = 0.13	$T_{26} = 1.4$<br>P > 0.05	**$T_{26} = 3.6$**<br>**P < 0.01**	**$T_{24} = 2.6$**<br>**P < 0.05**	$T_{26} = 0.36$<br>P > 0.05
		OA	**$F_{(1,60)} = 23.2$**<br>**P < 0.0001**	$F_{(1,60)} = 0.07$<br>P = 0.80	**$F_{(1,60)} = 15.7$**<br>**P < 0.001**	$T_{30} = 0.61$<br>P > 0.05	**$T_{30} = 6.2$**<br>**P < 0.0001**	**$T_{30} = 2.6$**<br>**P < 0.05**	**$T_{30} = 3.0$**<br>**P < 0.01**
	Test 2 (T_2)	ED	$F_{(1,57)} = 2.0$<br>P = 0.16	$F_{(1,57)} = 0.7$<br>P = 0.40	$F_{(1,57)} = 0.22$<br>P = 0.64	$T_{30} = 1.4$<br>P > 0.05	$T_{27} = 0.7$<br>P > 0.05	$T_{28} = 0.3$<br>P > 0.05	$T_{29} = 0.9$<br>P > 0.05
		YA	**$F_{(1,46)} = 7.5$**<br>**P < 0.01**	$F_{(1,46)} = 0.32$<br>P = 0.57	$F_{(1,46)} = 0.01$<br>P = 0.93	$T_{25} = 2.0$<br>P > 0.05	$T_{21} = 1.9$<br>P > 0.05	$T_{22} = 0.45$<br>P > 0.05	$T_{24} = 0.35$<br>P > 0.05
		OA	$F_{(1,60)} = 1.1$<br>P =0.29	$F_{(1,60)} = 0.22$<br>P = 0.64	$F_{(1,60)} = 0.23$<br>P = 0.63	$T_{30} = 0.41$<br>P > 0.05	T_{30} 1.1<br>P > 0.05	$T_{30} = 0.01$<br>P > 0.05	$T_{30} = 0.66$<br>P > 0.05
Female	Test 1 (T_1)	ED	**$F_{(1,59)} = 15.1$**<br>**P < 0.001**	$F_{(1,59)} = 3.2$<br>P = 0.08	**$F_{(1,59)} = 8.5$**<br>**P < 0.01**	**$T_{30} = 4.8$**<br>**P < 0.0001**	$T_{29} = 0.7$<br>P > 0.05	**$T_{30} = 3.3$**<br>**P < 0.01**	$T_{29} = 0.8$<br>P > 0.05
		YA	**$F_{(1,59)} = 30.7$**<br>**P < 0.0001**	$F_{(1,59)} = 0.52$<br>P = 0.47	$F_{(1,59)} = 0.88$<br>P = 0.35	**$T_{29} = 3.2$**<br>**P < 0.01**	**$T_{30} = 4.6$**<br>**P < 0.0001**	$T_{30} = 0.15$<br>P > 0.05	$T_{29} = 1.2$<br>P > 0.05
		OA	**$F_{(1,60)} = 36.4$**<br>**P < 0.0001**	$F_{(1,60)} = 0.01$<br>P = 0.93	$F_{(1,60)} = 3.1$<br>P = 0.08	**$T_{30} = 3.0$**<br>**P < 0.01**	**$T_{30} = 5.5$**<br>**P < 0.0001**	$T_{30} = 1.3$<br>P > 0.05	$T_{30} = 1.2$<br>P > 0.05
	Test 2 (T_2)	ED	**$F_{(1,58)} = 12.5$**<br>**P < 0.001**	**$F_{(1,58)} = 24.2$**<br>**P < 0.0001**	**$F_{(1,58)} = 19.3$**<br>**P < 0.0001**	**$T_{30} = 5.7$**<br>**P < 0.0001**	$T_{28} = 0.6$<br>P > 0.05	**$T_{28} = 6.5$**<br>**P < 0.0001**	$T_{30} = 0.4$<br>P > 0.05
		YA	**$F_{(1,60)} = 9.9$**<br>**P < 0.01**	$F_{(1,60)} = 0.16$<br>P = 0.69	$F_{(1,60)} < 0.001$<br>P = 0.98	$T_{30} = 2.2$<br>P > 0.05	$T_{30} = 2.3$<br>P > 0.05	$T_{30} = 0.30$<br>P > 0.05	$T_{30} = 0.26$<br>P > 0.05
		OA	**$F_{(1,60)} = 8.1$**<br>**P < 0.01**	**$F_{(1,60)} = 5.8$**<br>**P < 0.05**	$F_{(1,60)} = 0.02$<br>P = 0.90	$T_{30} = 2.1$<br>P > 0.05	$T_{30} = 1.9$<br>P > 0.05	$T_{30} = 1.6$<br>P > 0.05	$T_{30} = 1.8$<br>P > 0.05

3. Two-way ANOVA: EET effects on genotype and ages

**Fig. 2b OFT Total Activity**		**Genotype Effects**	**Age Effects**	**Genotype X Age Interactions**	***Test 2 hoc* Bonferroni Test Age Effects**						***Test 2 hoc* Bonferroni Test Genotype Effects (WT vs KIV)**		
					**ED vs YA**		**ED vs OA**		**YA vs OA**				
					**WT**	**KIV**	**WT**	**KIV**	**WT**	**KIV**	**ED**	**YA**	**OA**
Male	Test 1	$F_{(1, 83)}$ = 0.10 P = 0.75	**$F_{(2, 83)}$ = 12.3 P < 0.001**	$F_{(2, 83)}$ = 2.4 P = 0.10	T_{26} = 1.3 P > 0.05	T_{28} = 1.6 P > 0.05	T_{27} = 2.4 P > 0.05	**T_{30} = 4.9 P < 0.001**	T_{27} = 1.0 P > 0.05	**T_{28} = 3.2 P < 0.05**	T_{28} = 1.1 P > 0.05	T_{26} = 0.95 P > 0.05	T_{29} = 1.6 P > 0.05
	Test 2	$F_{(1, 78)}$ = 0.65 P = 0.42	**$F_{(2, 78)}$ = 10.1 P < 0.001**	$F_{(2, 78)}$ = 1.5 P = 0.22	T_{22} = 0.24 P > 0.05	T_{26} = 2.2 P > 0.05	T_{28} = 2.0 P > 0.05	**T_{30} = 4.4 P < 0.001**	T_{24} = 1.5 P > 0.05	T_{26} = 1.9 P > 0.05	T_{28} = 0.94 P > 0.05	T_{21} = 0.90 P > 0.05	T_{30} = 1.4 P > 0.05
Female	Test 1	**$F_{(1, 84)}$ = 5.1 P < 0.05**	$F_{(2, 84)}$ = 2.6 P = 0.08	$F_{(2, 84)}$ = 2.6 P = 0.08	T_{28} = 0.22 P > 0.05	T_{28} = 1.5 P > 0.05	T_{27} = 0.01 P > 0.05	**T_{29} = 3.2 P < 0.05**	T_{27} = 0.21 P > 0.05	T_{28} = 1.7 P > 0.05	**T_{28} = 2.8 P < 0.05**	T_{28} = 1.5 P > 0.05	T_{28} = 0.40 P > 0.05
	Test 2	**$F_{(1, 83)}$ = 14.4 P < 0.001**	$F_{(2, 83)}$ = 1.5 P = 0.23	$F_{(2, 83)}$ = 3.0 P = 0.06	T_{28}= 2.2 P > 0.05	T_{28} = 0.56 P > 0.05	T_{13} = 1.2 P > 0.05	T_{13} = 2.0 P > 0.05	T_{13} = 0.97 P > 0.05	T_{13} = 1.4 P > 0.05	**T_{27} = 4.1 P < 0.001**	T_{29} = 1.5 P > 0.05	T_{28} = 0.98 P > 0.05

**Fig. 3b OFT Time in Center**		**Genotype Effects**	**Age Effects**	**Genotype X Age Interactions**	***Test 2 hoc* Bonferroni Test Age Effects**						***Test 2 hoc* Bonferroni Test Genotype Effects (WT vs KIV)**		
					**ED vs YA**		**ED vs OA**		**YA vs OA**				
					**WT**	**KIV**	**WT**	**KIV**	**WT**	**KIV**	**ED**	**YA**	**OA**
Male	Test 1	$F_{(1, 79)}$ = 4.0 P = 0.05	$F_{(2, 79)}$ = 0.90 P = 0.41	$F_{(2, 79)}$ = 0.77 P = 0.47	T_{23} = 0.31 P > 0.05	T_{26} = 1.1 P > 0.05	T_{28} = 0.16 P > 0.05	T_{27} = 1.8 P > 0.05	T_{25} = 0.47 P > 0.05	T_{26} = 0.49 P > 0.05	T_{28} = 0.17 P > 0.05	T_{21} = 1.5 P > 0.05	T_{30} = 1.8 P > 0.05
	Test 2	**$F_{(1, 75)}$ = 4.6 P < 0.05**	$F_{(2, 75)}$ = 0.92 P = 0.40	$F_{(2, 75)}$ = 1.8 P = 0.17	T_{22} = 2.0 P > 0.05	T_{24} = 0.65 P > 0.05	T_{27} = 1.8 P > 0.05	T_{30} = 0.04 P > 0.05	T_{23} = 0.38 P > 0.05	T_{24} = 0.68 P > 0.05	**T_{28} = 2.9 P < 0.05**	T_{19} = 0.03 P > 0.05	T_{29} = 1.2 P > 0.05
Female	Test 1	$F_{(1, 87)}$ = 0.29 P = 0.59	$F_{(2, 87)}$ = 1.9 P = 0.15	**$F_{(2, 87)}$ = 4.0 P < 0.05**	T_{30} = 0.53 P > 0.05	**T_{27} = 3.4 P < 0.01**	T_{30} = 1.3 P > 0.05	T_{29} = 1.9 P > 0.05	T_{30} = 0.74 P > 0.05	T_{27} = 1.6 P > 0.05	**T_{30} = 2.6 P < 0.05**	T_{27} = 1.1 P > 0.05	T_{30} = 0.52 P > 0.05
	Test 2	$F_{(1, 86)}$ = 0.69 P = 0.41	$F_{(2, 86)}$ = 0.91 P = 0.41	$F_{(2, 86)}$ = 0.61 P = 0.55	T_{26} = 1.7 P > 0.05	T_{30} = 0.16 P > 0.05	T_{26}= 1.0 P > 0.05	T_{30} = 0.28 P > 0.05	T_{30} = 0.71 P > 0.05	T_{30}= 0.11 P > 0.05	T_{26} = 0.29 P > 0.05	T_{30} = 1.3 P > 0.05	T_{30} = 0.49 P > 0.05

Fig. 4b TST Immobility Time		Genotype Effects	Age Effects	Genotype X Age Interactions	*Test 2 hoc* Bonferroni Test Age Effects						*Test 2 hoc* Bonferroni Test Genotype Effects (WT vs KIV)		
					ED vs YA		ED vs OA		YA vs OA				
					WT	KIV	WT	KIV	WT	KIV	ED	YA	OA
Male	Test 1	$F_{(1, 78)}$ = 0.05<br>P = 0.82	$F_{(2, 78)}$ = 2.8<br>P = 0.06	**$F_{(2, 78)}$ = 3.6<br>P < 0.05**	T_{23} = 1.4<br>P > 0.05	T_{24} = 2.2<br>P > 0.05	T_{26} = 0.06<br>P > 0.05	**T_{26} = 3.1<br>P < 0.05**	T_{27} = 1.5<br>P > 0.05	T_{28} = 0.89<br>P > 0.05	T_{22} = 2.0<br>P > 0.05	T_{25} = 1.6<br>P > 0.05	T_{30} = 1.0<br>P > 0.05
	Test 2	$F_{(1, 72)}$ = 0.25<br>P = 0.62	**$F_{(2, 72)}$ = 4.7<br>P < 0.05**	**$F_{(2, 72)}$ = 7.3<br>P < 0.001**	T_{21} = 2.1<br>P > 0.05	T_{21} = 2.1<br>P > 0.05	T_{26} = 0.6<br>P > 0.05	**T_{25}= 3.5<br>P < 0.01**	T_{25} = 1.6<br>P > 0.05	T_{26} = 1.4<br>P > 0.05	**T_{22} = 2.8<br>P < 0.05**	T_{30} = 2.2<br>P > 0.05	T_{30} = 1.6<br>P > 0.05
Female	Test 1	$F_{(1, 90)}$ = 0.39<br>P = 0.53	**$F_{(2, 90)}$ = 6.6<br>P < 0.005**	$F_{(2, 90)}$ = 1.3<br>P = 0.27	T_{29} = 1.0<br>P > 0.05	T_{30} = 1.6<br>P > 0.05	T_{29} = 1.5<br>P > 0.05	**T_{29} = 3.5<br>P < 0.01**	T_{30} = 0.40<br>P > 0.05	T_{30} = 2.0<br>P > 0.05	T_{29} = 1.2<br>P > 0.05	T_{30} = 0.76<br>P > 0.05	T_{30} = 0.95<br>P > 0.05
	Test 2	**$F_{(1, 85)}$ = 6.3<br>P < 0.01**	$F_{(2, 85)}$ = 2.4<br>P = 0.10	**$F_{(2, 85)}$ = 4.1<br>P < 0.02**	T_{26} = 1.6<br>P > 0.05	T_{29} = 2.2<br>P > 0.05	T_{26} = 0.15<br>P > 0.05	**T_{29} = 3.1<br>P < 0.05**	T_{30} = 1.6<br>P > 0.05	T_{30} = 0.92<br>P > 0.05	**T_{26} = 3.6<br>P < 0.01**	T_{34}= 0.05<br>P > 0.05	T_{30} = 0.59<br>P > 0.05

Fig. 5b BDNF levels		Genotype Effects	Age Effects	Genotype X Age Interactions	*Test 2 hoc* Bonferroni Test Age Effects						*Test 2 hoc* Bonferroni Test Genotype Effects (WT vs KIV)		
					ED vs YA		ED vs OA		YA vs OA				
					WT	KIV	WT	KIV	WT	KIV	ED	YA	OA
Hippocampus	Test 1	$F_{(1, 45)}$ = 0.0002<br>P = 0.99	**$F_{(2, 45)}$ = 7.1<br>P < 0.005**	$F_{(2, 45)}$ = 1.5<br>P = 0.23	T_{16} = 0.67<br>P > 0.05	**T_{15} = 3.1<br>P < 0.05**	T_{16} = 1.9<br>P > 0.05	**T_{15} = 3.1<br>P < 0.05**	T_{14} = 1.2<br>P > 0.05	T_{14} = 0.01<br>P > 0.05	T_{17} = 1.3<br>P > 0.05	T_{14} = 1.2<br>P > 0.05	T_{14} < 0.001<br>P > 0.05
	Test 2	$F_{(1, 46)}$ = 3.6<br>P = 0.06	**$F_{(2, 46)}$ = 6.4<br>P < 0.005**	**$F_{(2, 46)}$ = 3.4<br>P < 0.05**	T_{16} = 0.48<br>P > 0.05	T_{16} = 2.3<br>P > 0.05	**T_{15} = 3.0<br>P < 0.05**	T_{16} = 2.0<br>P > 0.05	**T_{13} = 3.3<br>P < 0.05**	T_{14} = 0.25<br>P > 0.05	**T_{18} = 2.0<br>P < 0.05**	T_{14} = 1.1<br>P > 0.05	T_{13} = 1.9<br>P > 0.05
Frontal Cortex	Test 1	$F_{(1, 46)}$ = 0.30<br>P = 0.58	**$F_{(2, 46)}$ = 6.0<br>P < 0.005**	$F_{(2, 46)}$ = 0.62<br>P = 0.45	T_{16} = 1.0<br>P > 0.05	T_{15} = 2.6<br>P > 0.05	T_{16} = 2.0<br>P > 0.05	T_{16} = 2.6<br>P > 0.05	T_{14} = 0.93<br>P > 0.05	T_{13} = 0.03<br>P > 0.05	T_{18} = 1.1<br>P > 0.05	T_{13} = 0.61<br>P > 0.05	T_{14} = 0.45<br>P > 0.05
	Test 2	$F_{(1, 48)}$ = 0.02<br>P = 0.88	$F_{(2, 48)}$ = 0.72<br>P = 0.49	$F_{(2, 48)}$ = 0.42<br>P = 0.66	T_{16} = 0.12<br>P > 0.05	T_{15} = 0.26<br>P > 0.05	T_{15} = 1.4<br>P > 0.05	T_{15} = 0.28<br>P > 0.05	T_{13} = 1.2<br>P > 0.05	T_{12} = 0.03<br>P > 0.05	T_{18} = 0.23<br>P > 0.05	T_{13} = 0.22<br>P > 0.05	T_{12} = 0.79<br>P > 0.05

Suppl. Fig. 1b OFT Distance Moved		Genotype Effects	Age Effects	Genotype X Age Interactions	***Test 2 hoc* Bonferroni Test Age Effects**						***Test 2 hoc* Bonferroni Test Genotype Effects (WT vs KIV)**		
					**ED vs YA**		**ED vs OA**		**YA vs OA**				
					**WT**	**KIV**	**WT**	**KIV**	**WT**	**KIV**	**ED**	**YA**	**OA**
Male	Test 1	**$F_{(1, 83)}$ = 19.7 P < 0.001**	$F_{(2, 83)}$ = 4.8 **P < 0.05**	$F_{(2, 83)}$ = 3.2 P = 0.05	T_{26} = 0.02 P > 0.05	T_{27} = 0.87 P > 0.05	T_{28} = 0.34 P > 0.05	**T_{30} = 3.8 P < 0.01**	T_{28}= 0.36 P > 0.05	**T_{28} = 2.9 P < 0.05**	T_{27} = 1.1 P > 0.05	T_{26} = 2.0 P > 0.05	**T_{30} = 4.7 P < 0.001**
	Test 2	$F_{(1, 78)}$ = 0.26 P = 0.61	$F_{(2, 78)}$ = 2.6 P = 0.08	$F_{(2, 78)}$ = 1.6 P = 0.21	T_{23} = 0.13 P > 0.05	T_{25} = 0.64 P > 0.05	T_{28} = 0.26 P > 0.05	**T_{29} = 2.8 P < 0.05**	T_{25} = 0.38 P > 0.05	T_{26} = 2.0 P > 0.05	T_{27} = 0.78 P > 0.05	T_{20} = 0.03 P > 0.05	T_{30} = 1.7 P > 0.05
Female	Test 1	$F_{(1, 85)}$ = 0.08 P = 0.78	$F_{(2, 85)}$ = 0.87 P = 0.42	**$F_{(2, 85)}$ = 13.8 P < 0.001**	**T_{30} = 3.0 P < 0.05**	**T_{29} = 3.3 P < 0.01**	**T_{30} = 4.1 P < 0.001**	T_{30} = 2.6 P > 0.05	T_{30} = 1.1 P > 0.05	T_{30} = 0.79 P > 0.05	**T_{29} = 4.0 P < 0.001**	T_{30} = 2.3 P > 0.05	**T_{30} = 2.6 P < 0.05**
	Test 2	$F_{(1, 88)}$ = 3.5 P = 0.07	**$F_{(2, 88)}$ = 14.4 P < 0.001**	**$F_{(2, 88)}$ = 9.3 P < 0.001**	T_{28} = 0.78 P > 0.05	T_{30} = 0.83 P > 0.05	T_{28} = 1.9 P > 0.05	T_{30} = 0.55 P > 0.05	T_{30} = 1.1 P > 0.05	T_{30} = 0.29 P > 0.05	**T_{27}= 4.4 P < 0.001**	T_{30} = 0.05 P > 0.05	T_{30} = 1.4 P > 0.05

3. Student's *t*-test was conducted to compare data between two-groups. This test was to verify the results of two-way ANOVA and to avoid type II errors. No major discrepancy was found.

**Fig. 2 OFT (Total Activity)**			**Genotype Effect (WT vs KIV)**		**Treatment Effect (SCT vs EET)**	
			**SCT**	**EET**	**WT**	**KIV**
Male	ED	Test 1	**P < 0.001**	**P < 0.001**	**P < 0.001**	**P < 0.005**
		Test 2	**P < 0.001**	**P < 0.001**	**P < 0.005**	**P < 0.01**
	YA	Test 1	**P < 0.001**	**P < 0.001**	**P < 0.005**	**P < 0.05**
		Test 2	**P < 0.01**	**P < 0.001**	**P < 0.005**	P > 0.05
	OA	Test 1	**P < 0.01**	**P < 0.005**	**P < 0.05**	P > 0.05
		Test 2	**P < 0.005**	**P < 0.005**	P > 0.05	P > 0.05
Female	ED	Test 1	**P < 0.001**	P > 0.05	**P < 0.05**	**P < 0.001**
		Test 2	**P < 0.001**	P > 0.05	P > 0.05	**P < 0.001**
	YA	Test 1	**P < 0.001**	**P < 0.005**	**P < 0.05**	**P < 0.001**
		Test 2	**P < 0.001**	**P < 0.001**	**P < 0.05**	**P < 0.01**
	OA	Test 1	**P < 0.005**	**P < 0.005**	**P < 0.005**	P > 0.05
		Test 2	**P < 0.005**	**P < 0.01**	P > 0.05	P > 0.05

**Fig. 3 OFT (Time in Center)**			**Genotype Effect (WT vs KIV)**		**Treatment Effect (SCT vs EET)**	
			**SCT**	**EET**	**WT**	**KIV**
Male	ED	Test 1	P > 0.05	P > 0.05	**P < 0.01**	**P < 0.05**
		Test 2	P > 0.05	**P < 0.005**	**P < 0.05**	P > 0.05
	YA	Test 1	P > 0.05	**P < 0.005**	**P < 0.05**	P > 0.05
		Test 2	P > 0.05 (0.059)	P > 0.05	P > 0.05	P > 0.05
	OA	Test 1	P > 0.05	**P < 0.005**	**P < 0.05**	P > 0.05
		Test 2	P > 0.05	**P < 0.005**	P > 0.05	P > 0.05
Female	ED	Test 1	P > 0.05 (0.070)	P > 0.05	P > 0.05	**P < 0.01**
		Test 2	P > 0.05	P > 0.05	P > 0.05	P > 0.05
	YA	Test 1	P > 0.05	**P < 0.01**	P > 0.05	P > 0.05
		Test 2	P > 0.05	**P < 0.005**	P > 0.05	P > 0.05
	OA	Test 1	**P < 0.05**	**P < 0.005**	**P < 0.005**	P > 0.05
		Test 2	**P < 0.05**	**P < 0.05**	P > 0.05	P > 0.05

**Fig. 4 TST (Immobility Time)**			**Genotype Effect (WT vs KIV)**		**Treatment Effect (SCT vs EET)**	
			**SCT**	**EET**	**WT**	**KIV**
Male	ED	Test 1	**P < 0.001**	P > 0.05	**P < 0.005**	**P < 0.001**
		Test 2	**P < 0.001**	P > 0.05	P > 0.05	**P < 0.05**
	YA	Test 1	**P < 0.001**	**P < 0.005**	**P < 0.05**	**P < 0.001**
		Test 2	**P < 0.01**	**P < 0.01**	P > 0.05 (0.072)	P > 0.05
	OA	Test 1	**P < 0.05**	**P < 0.005**	**P < 0.05**	P > 0.05
		Test 2	P > 0.05	**P < 0.005**	P > 0.05	P > 0.05
Female	ED	Test 1	**P < 0.005**	P > 0.05	**P < 0.01**	**P < 0.001**
		Test 2	**P < 0.001**	P > 0.05	P > 0.05	**P < 0.05**
	YA	Test 1	**P < 0.001**	**P < 0.05**	**P < 0.05**	**P < 0.01**
		Test 2	**P < 0.01**	P > 0.05 (0.053)	P > 0.05	P > 0.05
	OA	Test 1	**P < 0.05**	**P < 0.005**	P > 0.05	P > 0.05
		Test 2	**P < 0.05**	P > 0.05	P > 0.05	P > 0.05

**Fig. 5 BDNF levels**			**Genotype Effect (WT vs KIV)**		**Treatment Effect (SCT vs EET)**	
			**SCT**	**EET**	**WT**	**KIV**
Hippocampus	ED	Test 1	**P < 0.001**	P > 0.05	**P < 0.001**	**P < 0.001**
		Test 2	**P < 0.001**	**P < 0.001**	P > 0.05	**P < 0.005**
	YA	Test 1	**P < 0.001**	**P < 0.001**	**P < 0.005**	P > 0.05
		Test 2	**P < 0.001**	**P < 0.001**	P > 0.05	P > 0.05
	OA	Test 1	**P < 0.001**	**P < 0.001**	P > 0.05	P > 0.05
		Test 2	**P < 0.01**	P > 0.05	P > 0.05	P > 0.05
Frontal Cortex	ED	Test 1	**P < 0.001**	**P < 0.05**	**P < 0.05**	**P < 0.005**
		Test 2	**P < 0.001**	**P < 0.001**	P > 0.05	P > 0.05
	YA	Test 1	**P < 0.005**	**P < 0.001**	P > 0.05	P > 0.05
		Test 2	**P < 0.005**	**P < 0.05**	P > 0.05	P > 0.05
	OA	Test 1	**P < 0.01**	P > 0.05	P > 0.05	P > 0.05
		Test 2	P > 0.05	P > 0.05	P > 0.05	P > 0.05

**Supplementary Fig. 1 OFT (Distance Moved)**			**Genotype Effect (WT vs KIV)**		**Treatment Effect (SCT vs EET)**	
			**SCT**	**EET**	**WT**	**KIV**
Male	ED	Test 1	P > 0.05	**P < 0.05**	**P < 0.05**	P > 0.05
		Test 2	P > 0.05	P > 0.05	P > 0.05	P > 0.05
	YA	Test 1	P > 0.05	**P < 0.005**	**P < 0.05**	P > 0.05
		Test 2	**P < 0.05**	P > 0.05	P > 0.05	P > 0.05
	OA	Test 1	P > 0.05	**P < 0.005**	**P < 0.05**	P > 0.05
		Test 2	P > 0.05	P > 0.05	**P < 0.005**	P > 0.05
Female	ED	Test 1	**P < 0.001**	P > 0.05	**P < 0.01**	P > 0.05
		Test 2	**P < 0.001**	P > 0.05	**P < 0.001**	P > 0.05
	YA	Test 1	**P < 0.005**	**P < 0.01**	P > 0.05	P > 0.05
		Test 2	**P < 0.05**	**P < 0.05**	P > 0.05	P > 0.05
	OA	Test 1	**P < 0.01**	**P < 0.005**	P > 0.05	P > 0.05
		Test 2	**P < 0.05**	P > 0.05	P > 0.05	**P < 0.05**
